# Genetic diversity of Bovine Viral Diarrhea Virus in cattle in France between 2018 and 2020

**DOI:** 10.3389/fvets.2022.1028866

**Published:** 2022-10-11

**Authors:** José Rivas, Alesia Hasanaj, Caroline Deblon, Philippe Gisbert, Mutien-Marie Garigliany

**Affiliations:** ^1^Fundamental and Applied Research for Animals and Health (FARAH), Faculty of Veterinary Medicine, University of Liège, Liège, Belgium; ^2^Ceva Santé Animale, Libourne, France

**Keywords:** Bovine Viral Diarrhea Virus, cattle, genetic diversity, genotype, France

## Abstract

Bovine Viral Diarrhea Virus (BVDV) is one of the main pathogens that affects ruminants worldwide, generating significant economic losses. Like other RNA viruses, BVDV is characterized by a high genetic variability, generating the emergence of new variants, and increasing the risk of new outbreaks. The last report on BVDV genotypes in France was in 2008, since which there have been no new information. The goal of this study is to determine the genetic diversity of BVDV strains currently circulating in France. To this aim, samples of cattle were taken from different departments that are part of the main areas of livestock production during the years 2018 to 2020. Using the partial sequence of the 5'UTR region of the viral genome, we identified and classified 145 samples corresponding to *Pestivirus A* and one sample corresponding to *Pestivirus D*. For the *Pestivirus A* samples, the 1e, 1b, 1d, and 1l genotypes, previously described in France, were identified. Next, the 1r and 1s genotypes, not previously described in the country, were detected. In addition, a new genotype was identified and was tentatively assigned as 1x genotype. These results indicate an increase in the genetic diversity of BVDV in France.

## Introduction

Bovine Viral Diarrhea Virus (BVDV) is a globally distributed cattle pathogen, which causes significant economic losses (1). This virus also affects other domestic animal species such as sheep, goat, camelids and swine (2), and wild animals including cervids and giraffes (3). BVDV is responsible for a wide spectrum of symptoms in cattle, which include immunosuppression, persistent infection, mucosal disease, respiratory syndromes and reproductive dysfunction, generating major economic losses for the cattle industry (4). BVDV is a member of the genus *Pestivirus* within the family *Flaviviridae*. This genus also includes other viruses affecting domestic livestock such as *Pestivirus C* also known as Classical Swine Fever Virus (CSFV) and *Pestivirus D* known as Border Disease Virus (BDV) (5). The BVDV genome consists of a single strand of positive-sense RNA (ssRNA+) ~12.3-kb-long, which is flanked by a 5′ untranslated region (UTR) and a 3′UTR. This genome encodes 11–12 structural and non-structural proteins (Npro, C, Erns, E1, E2, P7, NS2/3, NS4A, NS4B, NS5A, NS4B) (1). The 5′UTR being one of the most conserved genomic regions of BVDV, it is widely used for phylogenetic classification. Three main species of the genus *Pestivirus* have been identified in cattle: *Pestivirus A* (known as BVDV-1), which so far includes 23 genotypes (1a−1w), *Pestivirus B* (BVDV-2), including 4 genotypes (2a−2d) (6–8), and *Pestivirus H* (BVDV-3, also known as HoBi-like virus) for which 4 genotypes (3a−3d) have been described (8–10). A fourth species that affects cattle less frequently is *Pestivirus D*, also known as Border Disease Virus (BDV) (5). It mainly affects sheep, producing prenatal and postnatal infections in lambs. Interspecies transmission by *Pestivirus D* has been observed in cattle, goats, and pigs (11, 12).

Due to their ability or not to exhibit a cytopathogenic effect, BVDV isolates have been categorized into 2 biotypes, cytopathogenic (CP) and non-cytopathogenic (NCP) (1). *Pestivirus A* is the most widely distributed species globally, corresponding to 88% of the isolates worldwide (6). Within this species, several dominant genotypes have been described by region, such as 1a genotype in Africa, 1c in Australia, while in Asia, America and Europe 1b predominates. This high genetic variability is described throughout the worldwide distribution of the virus (6).

In the case of France, in the latest studies from 2001 and 2008, 73 viral strains were described, which were classified as 1e (*n* = 46) and 1b (*n* = 15) genotypes, the two dominant genotypes, followed by 1a (*n* = 3), 1d (*n* = 3) and 1l (*n* = 3). For the *Pestivirus B* species, only 3 sequences were identified (13, 14).

The *Pestivirus A* genotypes currently circulating in France are unknown. Pestiviruses are known for their rapid evolution that can lead to genetic drift. Here we provide an update on the phylogenetic classification of *Pestivirus A* strains recently collected from bovines throughout various regions of France.

## Materials and methods

### Sample collection

In this study, 211 samples of whole blood from cattle were analyzed, collected from 38 departments in France during the years 2018 to 2020, and confirmed as positive for BVDV by RT-PCR by departmental diagnostic laboratories or sent by veterinary practitioners after having confirmed the positivity of the animal by antigen detection on blood (Idexx SNAP BVDV, Westbrook, USA). The samples were sent to the laboratory of Pathology, Faculty of Veterinary Medicine, University of Liege, for further processing. The map of the geographical distribution of the samples was designed using the QGIS 3.24 software (15). The samples used in this study were obtained from Departmental diagnostic laboratories in the context of the surveillance plan against BVDV in France. Getting access to these samples did not require the study to be reviewed or approved by an Ethics Committee.

### RT-PCR, sequencing and phylogenetic analysis

The viral RNA was extracted from blood samples using NucleoSpin^®^ RNA kit (Macherey Nagel, Germany), following manufacturer's instructions. A 288-bp region of the region 5′UTR was amplified by end-point RT-PCR using the Pan-Pestivirus primers Forward 324: 5′-ATG CCC WTA GTA GGA CTA GCA-3′ and Reverse 326: 5′-TCA ACT CCA TGT GCC ATG TAC-3′ (14) and Luna Universal One-Step RT-qPCR Kit (New England BioLabs, USA). The results were visualized on a 1% agarose gel, amplicons of the expected size were purified using NucleoSpin Gel and PCR Clean-up kit^®^ (Macherey Nagel, Germany) and then sequenced by the Sanger method (Eurofins Genomics^®^). The sequences obtained were aligned using ClustalW, implemented in the Geneious 10.2.3 software (Biomatters, New Zealand). The phylogenetic tree of the 5′UTR region was built using the Maximum Likelihood method with the Kimura 2-parameter model, Gamma distribution with Invariant Sites, as determined by a model prediction analysis, and 1,000 bootstrap replicates using the MEGA X software (16). Reference sequences of *Pestivirus A, B*and *H*species and genotypes were retrieved from NCBI GenBank database and included in the phylogenetic analysis. Nucleotide similarity percentages were calculated using the distance matrix algorithm of the MEGA X software (16).

## Results

**Figure 1:**
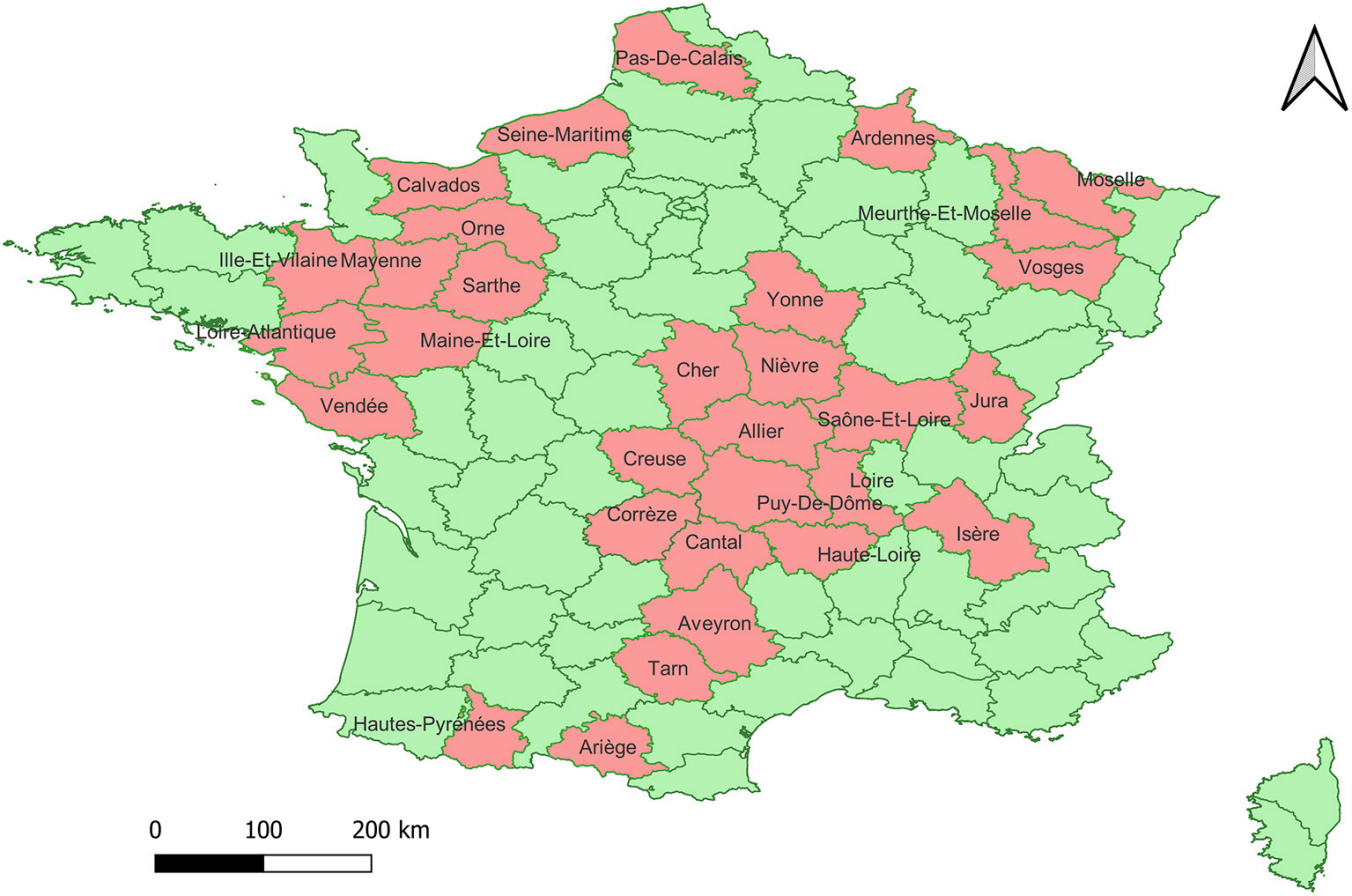
Map of the geographic distribution of the collection sites of the 146 samples included in the study. In pink the departments from which the samples have been collected.

Using a Pan-Pestivirus end-point RT-PCR targeting the 5′UTR it was possible to obtain an amplicon of the expected size from 146 of the analyzed samples, from 31 departments (Figure 1). The amplicons were purified and subsequently sequenced.

**Figure 2:**
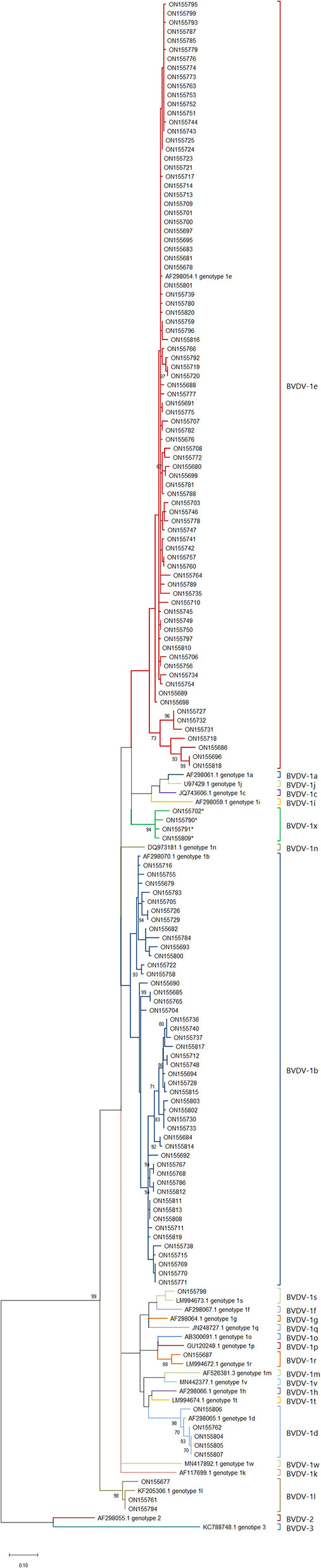
Phylogenetic tree of *Pestivirus A* genotypes circulating in France. Phylogenetic tree based on the BVDV 5′UTR fragment, using the Maximum Likelihood method and the two-parameter Kimura model (17). Reference strains were used for each known species/genotype. The GenBank accession number is indicated. Field strains described in this study are identified by the GenBank accession number (Table 1). The asterisk identifies the samples that correspond to the new 1x genotype.

**Figure 3:**
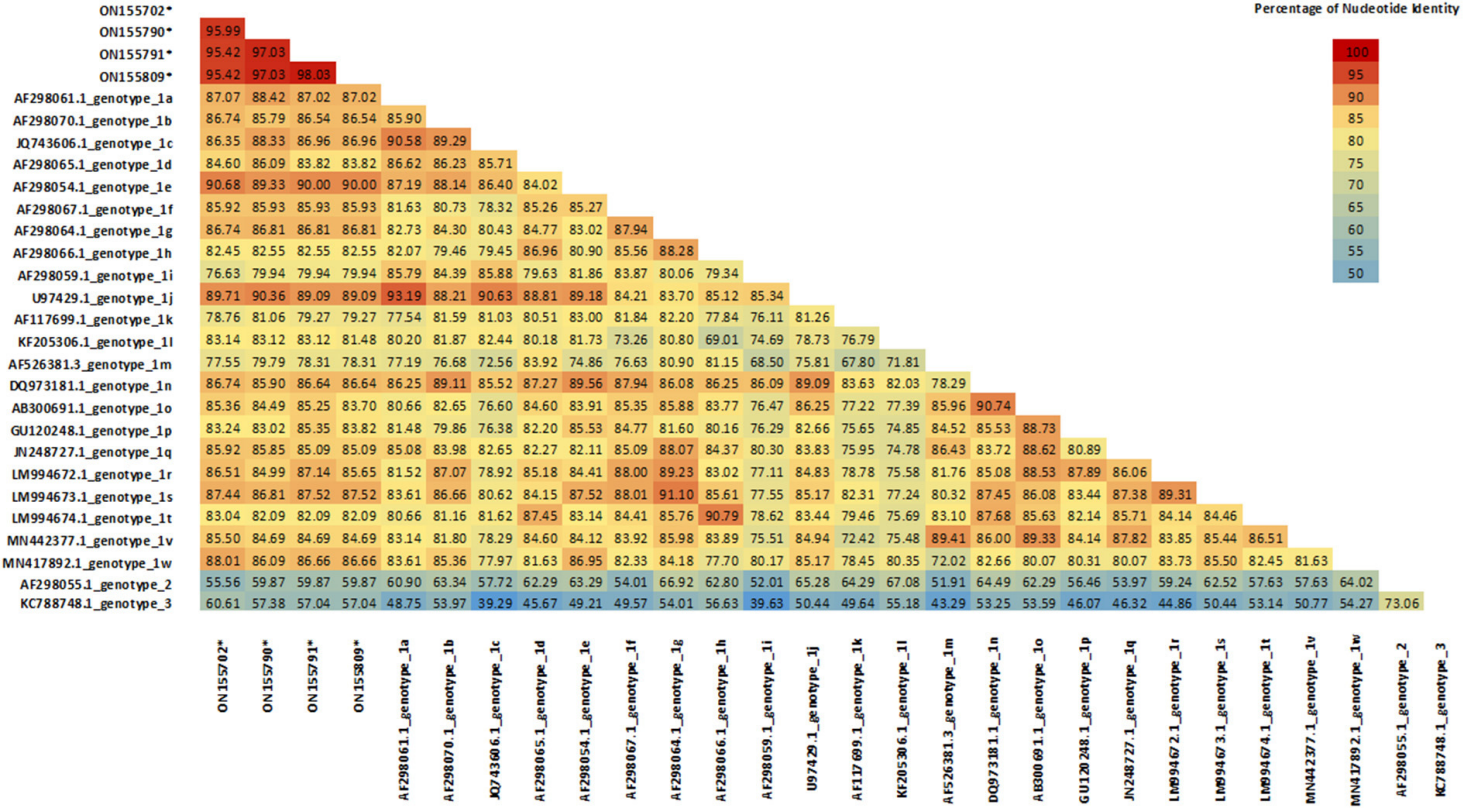
Sequence Identity Matrix of 1x strains compared with currently known *Pestivirus A* genotypes. Values generated from the 221-bp alignment of the 5′UTR segment. Sequences belonging to 1x genotype were compared with 24 reference strains of BVDV (one per known genotype).

These samples were compared with sequences from known genotypes of *Pestivirus A, B* and *H* species obtained from GenBank to build a phylogenetic tree. No sequence belonging to *Pestivirus B* and *H* were found. Except for sample ON693854, all the field sequences analyzed belonged to*Pestivirus A*. Most of them corresponded to genotypes 1e (57.9%) (*N* = 84) and to 1b (32.4%) (*N* = 47), being identified in 26 and 20 of the sampled departments, respectively. To a lesser extent, samples of the genotypes 1d (3.4%) (*N* = 5) and 1l (2.1%) (*N* = 3) were detected, each in 2 different departments. In addition, samples corresponding to 1s (0.7%) (*N* = 1) and 1r (0.7%) (*N* = 1) were identified for the first time in the country. Finally, 4 (2.7%) sequences from 3 different departments clustered together and separately from currently known genotypes within *Pestivirus A*, forming a new genotype tentatively named 1x (Figure 2). The nucleotide similarity of the 5'UTR is greater than 95.4% between the 1x sequences. The closest genotypes are 1e and 1j, sharing a similarity of 90.7 and 90.4%, respectively (Figure 3). In the phylogenetic analysis, sample ON693854 clustered outside the sequences corresponding to BVDV. Therefore, it was analyzed with other reference sequences of the *Pestivirus* genus, where it clustered within *Pestivirus D*species with sequences of the genotype 4 (data not shown).

The list of the samples analyzed is summarized in Table 1, where the distribution of the samples according to the department is detailed.

**Table 1:** List of field viral strains analyzed in this study.

**Accession number**	**Department**	**Genotype**	**Accession number**	**Department**	**Genotype**
ON155676	Yonne	1e	ON155749	Seine Maritime	1e
ON155677	Puy de Dôme	1l	ON155750	Seine Maritime	1e
ON155678	Puy de Dôme	1e	ON155751	Calvados	1e
ON155679	Puy de Dôme	1b	ON155752	Calvados	1e
ON155680	Cantal	1e	ON155753	Aveyron	1e
ON155681	Cantal	1e	ON155754	Vendée	1e
ON155682	Cantal	1b	ON155755	Meurthe et Moselle	1b
ON155683	Cantal	1e	ON155756	Meurthe et Moselle	1e
ON155684	Cantal	1b	ON155757	Meurthe et Moselle	1e
ON155685	Cantal	1b	ON155758	Meurthe et Moselle	1b
ON155686	Saône et Loire	1e	ON155759	Meurthe et Moselle	1e
ON155687	Vosges	1r	ON155760	Meurthe et Moselle	1e
ON155688	Mayenne	1e	ON155761	Meurthe et Moselle	1l
ON155689	Tarn	1e	ON155762	Vosges	1d
ON155690	Hautes Pyrénées	1b	ON155763	Cantal	1e
ON155691	Cantal	1e	ON155764	Aveyron	1e
ON155692	Tarn	1b	ON155765	Aveyron	1b
ON155693	Cher	1b	ON155766	Aveyron	1e
ON155694	Pas de Calais	1b	ON155767	Aveyron	1b
ON155695	Tarn	1e	ON155768	Aveyron	1b
ON155696	Ardennes	1e	ON155769	Aveyron	1b
ON155697	Pas de Calais	1e	ON155770	Aveyron	1b
ON155698	Tarn	1e	ON155771	Aveyron	1b
ON155699	Saône et Loire	1e	ON155772	Aveyron	1e
ON155700	Loire Atlantique	1e	ON155773	Aveyron	1e
ON155701	Loire Atlantique	1e	ON155774	Aveyron	1e
ON155702	Loire Atlantique	1x	ON155775	Aveyron	1e
ON155703	Loire Atlantique	1e	ON155776	Aveyron	1e
ON155704	Loire Atlantique	1b	ON155777	Aveyron	1e
ON155705	Loire Atlantique	1b	ON155778	Isère	1e
ON155706	Sarthe	1e	ON155779	Isère	1e
ON155707	Sarthe	1e	ON155780	Isère	1e
ON155708	Sarthe	1e	ON155781	Isère	1e
ON155709	Sarthe	1e	ON155782	Isère	1e
ON155710	Loire Atlantique	1e	ON155783	Isère	1b
ON155711	Maine et Loire	1b	ON155784	Isère	1b
ON155712	Maine et Loire	1b	ON155785	Isère	1e
ON155713	Loire Atlantique	1e	ON155786	Isère	1b
ON155714	Maine et Loire	1e	ON155787	Isère	1e
ON155715	Maine et Loire	1b	ON155788	Creuse	1e
ON155716	Maine et Loire	1b	ON155789	Creuse	1e
ON155717	Maine et Loire	1e	ON155790	Creuse	1x
ON155718	Maine et Loire	1e	ON155791	Creuse	1x
ON155719	Ariège	1e	ON155792	Corrèze	1e
ON155720	Ariège	1e	ON155793	Nièvre	1e
ON155721	Ariège	1e	ON155794	Meurthe et Moselle	1l
ON155722	Ariège	1b	ON155795	Meurthe et Moselle	1e
ON155723	Haute Loire	1e	ON155796	Meurthe et Moselle	1e
ON155724	Haute Loire	1e	ON155797	Allier	1e
ON155725	Haute Loire	1e	ON155798	Moselle	1s
ON155726	Ariège	1b	ON155799	Aveyron	1e
ON155727	Ariège	1e	ON155800	Vosges	1b
ON155728	Ariège	1b	ON155801	Tarn	1e
ON155729	Ariège	1b	ON155802	Tarn	1b
ON155730	Ariège	1b	ON155803	Tarn	1b
ON155731	Ariège	1e	ON155804	Nièvre	1d
ON155732	Ariège	1e	ON155805	Nièvre	1d
ON155733	Ariège	1b	ON155806	Nièvre	1d
ON155734	Allier	1e	ON155807	Nièvre	1d
ON155735	Mayenne	1e	ON155808	Nièvre	1b
ON155736	Mayenne	1b	ON155809	Jura	1x
ON155737	Mayenne	1b	ON155810	Jura	1e
ON155738	Mayenne	1b	ON155811	Saône et Loire	1b
ON155739	Mayenne	1e	ON155812	Allier	1b
ON155740	Mayenne	1b	ON155813	Saône et Loire	1b
ON155741	Mayenne	1e	ON155814	Nièvre	1b
ON155742	Mayenne	1e	ON155815	Ille et Vilaine	1b
ON155743	Orne	1e	ON155816	Ardennes	1e
ON155744	Moselle	1e	ON155817	Ardennes	1b
ON155745	Orne	1e	ON155818	Ardennes	1e
ON155746	Cantal	1e	ON155819	Ardennes	1b
ON155747	Orne	1e	ON155820	Ardennes	1e
ON155748	Loire	1b	ON693854	Meurthe et Moselle	BDV-4

The sample number, department and genotype are detailed.

## Discussion

In this study we evaluated the genetic diversity of BVDV strains currently circulating in France. Viral sequences amplified by RT-PCR were genotyped based on the analysis of the 5' UTR. Out of 211 samples initially detected as positive for BVDV in Departmental laboratories or by veterinary practitioners, only 146 proved positive using the 5′UTR RT-PCR in our laboratory and yielded sequences of sufficient quality for further analyses. The delay between the initial detection and the reception of the samples in our laboratory and poor preservation conditions (samples stored at −20°C in the field laboratories, freeze-thaw cycles) likely contributed to explain such a difference.

The positive samples came from 31 departments that are part of the livestock production area with the highest numbers of animals per department in France (18). The samples corresponding to the *Pestivirus A* species were classified into 7 genotypes, in order of abundance: 1e, 1b, 1d, 1x, 1l, 1r and 1s. In previous studies carried out in 2001 and 2008, the circulation of the genotypes 1e (*N* = 46), 1b (*N* = 15), 1a (*N* = 3), 1d (*N* = 3), 1l (*N* = 3) and *Pestivirus B* genotype 2a (*N* = 3) genotypes was detected in France (13, 14). Our data confirm that, as previously reported, 1e and 1b are the most abundant genotypes in France (13, 14).

Between 2001 and 2008 1e was the dominant genotype in France, representing 65% of the strains identified (13, 14). In our study the distribution remained in the ranges previously observed, decreasing slightly to 57.9% among the strains identified. The 1e genotype has been described mostly in Europe. In America 1e isolates have been reported in cattle in Brazil (19) and recently in Argentina (20). In Europe 1e genotype is distributed throughout the entire continent, mainly in France, where it is the dominant strain (13) and in Italy (21) and Switzerland (22), where it is the second most abundant strain. The 1e genotype is estimated to have originated in northern Italy in the Lombardy region between 1957 and 1988, where a large number of isolates are concentrated (23). From this region it spread to the rest of the country and to neighboring countries between 1990 and 2000 following the movements of cattle (21). In Switzerland, the highest concentration of 1e is in the west of the country, bordering the Lombardy region, associated with Felckvieh cattle (22). In France, the first cases of 1e were detected in 1993 (14). In this work, a geographic cluster of 1e was not observed. On the contrary, this genotype was found throughout the French territory, similar to the current propagation of this genotype in Italy (21).

The 1b genotype has a worldwide distribution, being the dominant genotype in America, Asia and most of Europe. In the latter it is the dominant strain in Italy and Spain, while it is the second most abundant genotype in France as in Germany (6). Until 2008, 21.4% of the samples identified in France corresponded to 1b (13, 14). In the current study this percentage increased to 32.4% of the samples. This rising trend has been described elsewhere over recent years for this genotype (24, 25).

The 1d genotype was identified with a lower frequency. This strain has recently been described in China (7) and Brazil (26). In Europe it is distributed throughout the continent, mainly in Sweden, where it is dominant (6). The 1d genotype had previously been described in France with a low presence of 4.3% in 2008 (13), as in this work, a frequency of 3.4% was observed for 1d, without great variation over time.

The 1l genotype is mainly found in Turkey, where it is the most abundant genotype, but it has also been identified in France, Italy and Spain with a very low proportion (6). In this study, only three sequences were identified as 1l corresponding to 2.1%, maintaining the same relative abundance as in previous studies where a frequency of 4.3% was observed (13).

Z-TEST statistical one-tailed analysis of the proportions of genotypes 1e, 1b, 1d and 1l between the previous studies in France (13, 14) and the current study, indicate that only the increase in genotype 1b represents a significant change (alpha < 0.05).

For the first time in France, the 1r and 1s genotypes were detected. 1r genotype has been described in Italy, Turkey (6, 27) and Poland (28), while 1s was described in Italy (27) and Poland (28). These genotypes are rare and show a low prevalence in the countries where they have been described (6, 28).

In this work, a new genotype formed by the sequences ON155702, ON155790, ON155791 and ON155809 was identified. These sequences have a nucleotide similarity >95.5% between them. With the closest genotypes 1e and 1j, the similarity was 90.7 and 90.4%, respectively. These percentages of similarity are within the ranges observed between currently known genotypes (14), suggesting that this cluster forms a new genotype different from those previously described. We decided to tentatively assign the name 1x to this new genotype, which follows 1w, the last genotype described (7). Although 1x genotype was previously used in Switzerland to describe the strain CH-01-08 in 2008 (29), this strain was finally reclassified as 1l (30). The appearance of new genotypes is an event that is classically observed with the evolution of BVDV. In China for example, two new genotypes 1v and 1w were recently described (7, 31). This is mainly attributed to the lack of proofreading function of the RNA polymerase during BVDV genome replication. This variability leads to a genetic drift, generating new genotypes and species over time (32).

In contrast, the 1a genotype, previously present in France, was not identified in this work. This genotype is the second most abundant globally, has been isolated on all continents and is used in most vaccines. In Europe it is mainly found in Ireland and the United Kingdom (6, 33). In France it was only identified in 2001 (14), but neither in the 2008 study (13), nor in the current study.

In this work, no strains belonging to the *Pestivirus B* species were identified. The latest reports are from 1994 with 2 samples typed as *Pestivirus B* genotype 2a (14, 34) and 2008, with a sample from Nièvre typed as *Pestivirus B* (13). These samples represented 1.3% of the 218 BVDV samples previously identified in the country (13, 14). The absence of this species in this work and the low frequency in previous studies suggest that the presence of *Pestivirus B* in France is sporadic as seen in Italy (35) or that it has disappeared.

Sequences belonging to the *Pestivirus H* species were not detected either. This emerging group was detected for the first time in contaminated fetal bovine serum from Brazil, since then it has been detected in America, Asia and Europe (36). So far in Europe, it has only been detected in Italy associated with an outbreak of abortions in 2011 (37).

In addition, in this work, the sample ON693854 was classified as *Pestivirus D* genotype 4. This sample was obtained from a calf belonging to a BVDV-free farm where cattle and sheep herds were kept together. Interestingly, the infection coincides with the recent introduction of sheep to the farm from the South of France. Cross-species infection is common in *Pestivirus D* and has been documented in cows housed with sheep in the same barn or on pasture (38). In France, *Pestivirus D* has been previously described, with 3, 4, 5, 6 and Tunisian and Tunisian-like genotypes (39–41). The genotype 4 is the dominant in sheep flocks in Spain (12, 42), and has been associated with several outbreaks in chamois (*Rupicapra pyrenaica pyrenaica*) in the Pyrenees throughout Spain, France and Andorra (40). To our knowledge, *Pestivirus D* genotype 4 infection in cattle has only been evidenced by viral neutralization test (43), so this could be the first case of molecular characterization of genotype 4 in cattle documented in the literature.

Since 2020, a decree has made it mandatory to monitor and control BVDV throughout France in order to eradicate it. Screening is based on individual virus search on all calves before 20 days of age or serological monitoring of bulk tank milk or blood from a sample of randomly selected animals. When analysis results are not favorable, the herd is declared BVDV infected and sanitary measures are implemented. In particular, the testing and elimination of all PI confirmed animals (44).

In France between 1982 and 2015, according to online data from the national food safety agency (ANSES) (45), 11 vaccines, including three with the fetal protection claim, have been approved for use in cattle against BVDV. These are composed of mono- and polyvalent vaccines with inactivated or modified live viruses (MLV) of genotypes 1a, 1b and 2a (45). The humoral protection of vaccines against homologous genotypes is well documented and is superior to that against heterologous genotypes (25, 46–48). In the case of the most abundant genotype in this study, i.e., the 1e genotype, cross-reactivity tests with vaccines composed of 1a and 1b genotypes and a strain belonging to this genotype have shown a complete absence of neutralizing antibodies (48), possibly due to the significant antigenic differences against this strain (29, 49). Nevertheless, data are not sufficient to generalize this finding to all the BVD-1e strains. In the case of the 1b genotype, cross-protection studies with vaccines containing 1a showed contradictory results. While some studies showed high levels of cross-reactivity (46, 48, 50), other studies suggested moderate to insufficient protection against 1b genotype (47, 48, 51, 52). This could be due to the high diversity observed in the 1b genotype and the use of 5′UTRs for phylogenetic classification rather than key viral antigenic protein sequences (25).

On the other hand, a protective cellular immune response triggered by vaccines can occur even before or without the presence of antibodies (53–55). This response is mostly observed with MLV. Unlike inactivated vaccines, MLV have the ability to replicate in the cytoplasm and synthesize viral proteins, such as NS2/3 (56) and NS3, that are highly conserved among *Pestiviruses A, B, H* (57). These proteins generate reactivity of CD4+, CD8+ T cells, which is speculated to be responsible for the good “inter-genotype” cross-protection conferred by *Pestivirus A* vaccines (57).

T-cell and B-cell response levels approaching sterilizing immunity are required for the prevention of fetal infections (32), which are mostly observed in animals vaccinated with MLV. In studies of animals challenged with BVDV, those that were vaccinated with MLV presented greater fetal protection (50, 58, 59) even against other species such as *Pestivirus H* (60), compared to inactivated vaccines that present variable levels of protection (51) or lack of protection from fetal infection, as it has been reported in recent studies (61, 62).

Due to the great genetic diversity of BVDV and the constant appearance of new genotypes, it is important to consider, in the application of eradication plans, the use of vaccines that generate proven cross-protection against heterologous strains present in the country. Moreover, these new genotypes can also affect the diagnosis of *Pestiviruses*, as it was the case for *Pestivirus H*, for which it was necessary to develop a new RT-qPCR assay to increase the detection efficiency of this species (63). For these reasons, it is important to maintain regular follow-up of the genetic evolution of BVDV, allowing the implementation of adequate eradication plans, as well as their monitoring to effectively control the disease.

In summary, in this work the genetic diversity of BVDV in France was updated, showing an increase in the variability of *Pestivirus A* strains. Only genotypes belonging to species *Pestivirus A* were found in circulation, the most abundant being 1e and 1b. Furthermore, genotypes 1r, 1s and 1x are described for the first time in France, the latter being a new genotype.

## Data availability statement

The datasets presented in this study can be found in online repositories. The names of the repository/repositories and accession number(s) can be found in the article/supplementary material.

## Ethics statement

The samples used in this study were obtained from Departmental Diagnostic Laboratories in the context of the surveillance plan against BVDV in France. Getting access to these samples did not require the study to be reviewed or approved by an Ethics Committee.

## Author contributions

JR participated to the laboratory analyses and wrote the original draft. CD helped in the construction of figures. AH was the technical support for the RNA extractions and RT-PCR. M-MG and PG designed the study and revised the manuscript. All authors contributed to the article and approved the submitted version.

## Funding

This research was conducted between Ceva Santé Animale and the Department of Pathology of the FMV of the University of Liège without specific funding.

## Conflict of interest

Author PG was employed by Ceva Santé Animale. The authors declare that this study received funding from Ceva Santé Animale. The funder was not involved in the laboratory work, analysis and interpretation of the data.

## Publisher's note

All claims expressed in this article are solely those of the authors and do not necessarily represent those of their affiliated organizations, or those of the publisher, the editors and the reviewers. Any product that may be evaluated in this article, or claim that may be made by its manufacturer, is not guaranteed or endorsed by the publisher.

## Abbreviations

BDV: Border Disease Virus
BVDV: Bovine Viral Diarrhea Virus
CP: Cytopathogenic
CSFV: Classical Swine Fever Virus
MLV: modified live viruses
NCP: non-cytopathogenic
ssRNA+: single strand of positive-sense RNA
UTR: untranslated region.
